# Exercise instructors are not consistently implementing the strength component of the UK chief medical officers’ physical activity guidelines in their exercise prescription for older adults

**DOI:** 10.1186/s12889-023-17289-w

**Published:** 2023-12-06

**Authors:** Ashley Gluchowski, Helena Bilsborough, Jane McDermott, Helen Hawley-Hague, Chris Todd

**Affiliations:** 1 School of Health & Society, University of Salford, Salford, UK; 2 Healthy Ageing Research Group, School of Health Sciences, University of Manchester, Manchester, UK; 3 National Institute for Health and Care Research, Applied Research Collaboration-Greater Manchester, School of Health Sciences, University of Manchester, Manchester, UK

**Keywords:** Ageing, Resistance training, Government, Policy, Recommendations

## Abstract

Strength training recommendations have been embedded within the UK’s Chief Medical Officers’ physical activity guidelines since 2011. There is limited evidence that these recommendations are used by exercise instructors in the community to underpin strength training prescription in the older adult population. This study aimed to explore exercise instructors’ awareness and utilisation of the guidelines when prescribing strength training to older adults. Fifteen exercise instructors working with older adults in the UK participated in one online interview. A general inductive approach was conducted and thematic analysis allowed for major themes to be identified from the raw data. We found that most exercise instructors (n = 9), but not all (n = 6), were aware of the guidelines. Only one instructor (n = 1) had reportedly implemented the guidelines into their practice; other instructors reported that the guidelines were irrelevant. Instead, each of the instructors had their preferred sources of information that they relied on to underpin their exercise prescription, and each had their own interpretation of ‘evidence-based strength training.’ This individualised interpretation resulted in exceptionally varied prescription in the community and does not necessarily align with the progressive, evidence-based prescription known to build muscular strength. We suggest that (i) more detail on *how* to build muscular strength be embedded within the guidelines, (ii) a handbook on how to implement the guidelines be made available, (iii) theoretical and practical teaching materials and courses be updated, and/or (iv) a re-(education) of exercise instructors already in the field may be necessary to bring about a consistent, evidence-based strength prescription necessary for the best possible health and longevity outcomes for our ageing population.

## Introduction

Higher levels of muscular strength have been shown to be associated with a lowered risk of age-associated disease including dementia [1], stroke [2], heart failure [3], as well as all-cause mortality [4, 5]. One of the most effective ways to build muscular strength is through progressively overloading the muscle, as in progressive resistance training (PRT) [6, 7]. Several systematic reviews and meta-analyses have definitively shown that older adults retain their ability to gain substantial increases in muscular strength with PRT [8–11].

**Fig. 1:**
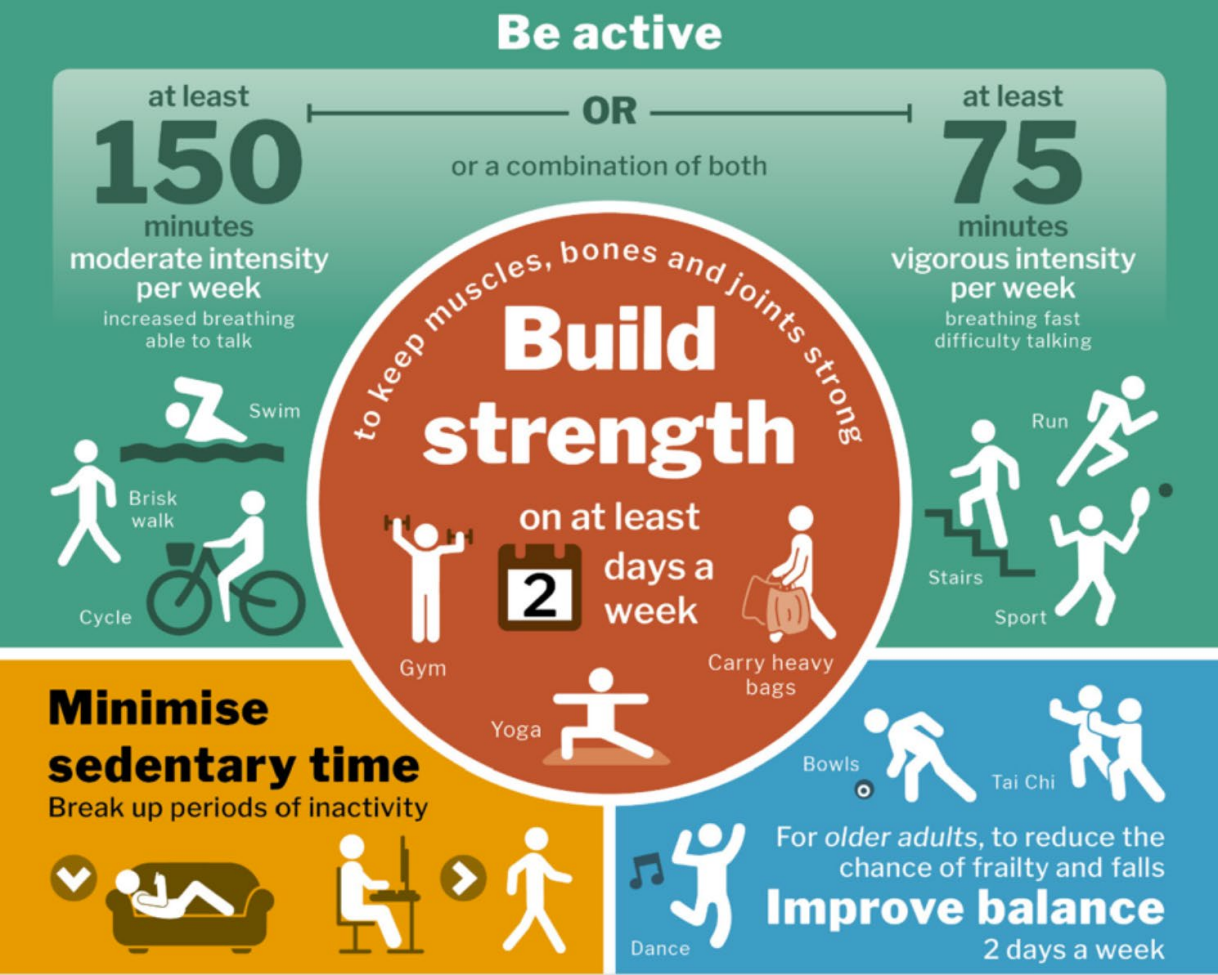
The 2019 United Kingdom’s Chief Medical Officers’ physical activity guidelines infographic (page 35) [12]

In 2011, as a result of the compelling literature in this area, the United Kingdom (UK) developed population-wide physical activity recommendations, known as the Chief Medical Officers’ physical activity guidelines (CMO PAG). Updated in 2019, the 65-page report from the older adult section (65 years and over) indicates that *‘older adults should maintain or improve their physical function by undertaking activities aimed at improving or maintaining muscle, balance, and flexibility on at least two days a week’* (p40) [12]. In the (presumably) public-facing infographic, the aerobic guidelines encourage adults and older adults to participate in ‘at least 150 minutes of moderate intensity activity per week or at least 75 minutes of vigorous intensity activity per week’ and is illustrated with examples of swimming, brisk walking, cycling, running, climbing stairs, and playing sport. On that same infographic, the strength component encourages adults and older adults to ‘*build strength on at least 2 days a week*’ and is illustrated with a person with dumbbells in each hand at the gym, a person in a yoga pose, and a person carrying heavy bags (Fig. 1). Thus, the reader is provided guidance on the recommended frequency, intensity, time, and type for aerobic prescription and the frequency and type for their strength prescription (notably omitting intensity and time) [12].

While the general population, and older adults specifically, are not meeting the aerobic guidelines, we (and others) have found that even fewer older adults meet the strength guidelines [13–16]. For instance, in the 2020–2021 Active Lives survey by Sport England, approximately 60% of adults 55–74 years of age reported to be participating in 150 min of physical activity per week, while only 42% reported to be participating in muscle strengthening activity twice per week [14]. Older adults as well as physiotherapists and health care professionals have all been found to be generally unaware of the physical activity guidelines in the UK with little evidence of implementation or embedment into their respective practices [15, 17–21]. There is much less evidence into the awareness or implementation of the strength component. Perhaps not surprising, as physical activity promotion has not historically been the responsibility of the physiotherapist or the medical community. Physiotherapists have been educated in and are focused on short-term restoration of physical function [18]. General practitioners have been trained to prescribe pharmaceuticals for the treatment of disease and as a result, report to feel far less confident discussing and prescribing exercise [19]. The general older adult public typically have neither a clinical, medical, or an exercise science background.

On the other hand, there are a more heterogenous sets of professionals (exercise instructors (level 3), personal trainers, clinical exercise physiologists) who have been specifically educated in exercise prescription, who work with the older adult population, and are responsible for discussing, promoting, prescribing, supervising, and motivating their clients during exercise. Resistance training should be a major component of what this group of exercise professionals prescribes, given its well-established benefits on the ageing musculoskeletal system [7]. Yet even the reliable use of resistance training in clinical and professional practice has recently come into question [22–24].

Therefore, while our previous study has shown that older adults themselves are not aware, nor participating in levels of strength training that would meet the UK’s CMO PAG [17], the current study aims to explore whether the exercise instructors who work with older adults are aware of and aligning their strength training prescription to the recommendations within the Chief Medical Officers’ physical activity guidelines.

## Methods

The authors took a qualitative approach, with in-depth semi-structured, one-on-one online interviews with a convenience sample conducted by the lead author (AG).

Advertisements for the study were included in e-newsletters sent from ageing organisations (Active Ageing Lead via Greater Sport, Greater Manchester Ageing Hub, Ambition for Ageing) and in a Facebook group for exercise instructors in the UK. Twenty-one people contacted the lead author via e-mail expressing interest *and* self-identified as an exercise instructor (prescribed and supervised exercise for older adult clients as part of their day-to-day work) in the UK. Fifteen people provided their written informed consent and were subsequently interviewed for this study (from 11/2021 to 12/2021) (See Table 1 for participant demographics).

**Table 1:** Participant demographics

		n=
Sex	Females Males	11 4
Age	18–24 years 25–34 years 35–44 years 45–54 years 55–64 years 65–74 years	0 1 4 1 8 1
Experience	< 1 year 1–4 years 5–10 years > 10 years	1 5 5 4

There was no prior relationship to any of the interviewees and interviewees were not given any knowledge about the lead researcher or reasons for doing the research until after the interviews were complete. This was in an attempt to prevent the interviewees from coming into the interview with pre-conceived ‘answers.’

Demographic data was collected with the first question during the one-to-one interviews as, ‘*please tell me a little about yourself - your age, your education and qualifications and experience in working with older adults.*’ Interviewees ranged in age from 29 to 71 years, with a mean±SD age of 52±12 years. Five interviewees were younger than 45 years old, while the remaining (n = 10) were in their mid-to-late 50s (n = 6), 60s (n = 3) and early 70s (n = 1). All interviewees were living and working in the UK at the time of their interview. Interviewees self-identified with ‘prescribing exercise to older adults’ as part of their job description. Interviewees’ qualifications and experience varied considerably.

The interview questions are shown in Table 2; impromptu follow-up questions allowed the lead author to further explore interviewees’ responses. Interviews lasted approximately 30-minutes and took place on and recorded using the online platform, Zoom. The audio recording was then transcribed verbatim. Transcripts were offered to interviewees for comment or correction. Five of 15 interviewees took up this offer and no major corrections were made. Transcripts were coded using NVivo 12 (qrsinternational.com). A £25 online gift card was offered as a small token of appreciation following the interview.

**Table 2:** Interview questions

1. Can you tell me a bit about your education, qualifications, and experience with older adults?
2. Can you tell me what informs your exercise prescription? Example follow up: For example, do you utilise guidelines or research in your exercise prescription? Example follow up: You mentioned you have clients with [health condition]. How do you know what to exercises to prescribe for those clients?
3. Are you aware of the 2019 Chief Medical Officers’ physical activity guidelines? If yes, can you describe them?
4. Do you use the Chief Medical Officers’ physical activity guidelines in your practice? Why or why not? Example follow up: Can you tell me more about the reaction you receive when you discuss participating in strength training twice per week? Example follow up: What information would be more helpful to you as an instructor?

The study used a general inductive approach and reflexive thematic analysis to develop and describe core narratives shared across the dataset [25, 26]. The lead author read and re-read each of the transcripts to immerse herself further in the data. Next, codes were applied to segments of the data that included points relevant to the study’s aims. Codes were then arranged and rearranged to form building blocks or themes found across transcripts [25]. A second author (HB), independently analysed a random subset of transcripts, followed by in-depth discussion and note taking to help ensure reflection and clarity in the first authors’ interpretation and naming of themes (see Table 3) [26].

## Results

Three major themes with nine subthemes were identified in the data (see Table 3).

**Table 3:** Major themes and main sub-themes in the data

Major Themes	Main Sub-Themes
The strength component within the Chief Medical Officers’ physical activity guidelines	Awareness and knowledge
	Implementation into practice
	Suggestions for improvement to the guidelines
Building strength	Benefits of building strength
	Reactions to building strength
	Reflections
The interpretation of evidence-based strength training	A more engaging model than strength training?
	Misconceptions of strength training
	Ageism in the implementation of strength training

### Major theme 1 – strength component within the chief medical officers’ physical activity guidelines (CMO PAG)

#### Main sub-theme 1a – awareness and knowledge

Nine of the 15 exercise instructors indicated that they were aware of the CMO PAG, ‘*We’ve got, we’ve got very aware of the guidelines’ (Female, aged 43; hereinafter, F, 43, qualified physiotherapist, level 4 postural stability instructor, level 4 cardiac and pulmonary instructor)*. Six were less so, ‘*the physical activity guidelines? There are lots of guidelines’ (F, 35, level 4 Pilates, exercise referral).*

We asked interviewees where they acquired their knowledge of exercise prescription for older adults. Nearly every interviewee described a different source, finding the information they needed on a diverse number of platforms. Information sources mentioned included UK Active, Sport England, Public Health England (PHE), the British Association for Cardiovascular Prevention and Rehabilitation (BACPR), the Chartered Institute for the Management of Sport and Physical Activity (CIMSPA), Later Life Training (LLT), So Much Improvement with a Little Exercise (SMILE), the news (British Broadcasting Corporation, BBC), and ‘*Dr. Google.’* One interviewee delivering Pilates classes to older adults specifically looked to the National Institute for Health and Care Excellence (NICE),

*At least if I follow them [NICE], then I can’t go wrong, because in the world of Pilates, there isn’t any guidance – well, there is guidance, but we have no sort of like governing body… (F, 53, level 3 older adults, exercise referral, trained as physiotherapist)*.

Interviewees also used social media like Facebook and Twitter,

*I get an awful lot of information off Twitter…I’m supposed to do CPD [Continuous Professional Development], which I’m a bit bad at. (F, 71*, *level 3 personal trainer)*

Some looked to colleagues and mentors for ‘ideas’,

*It’s a half and half thing. Half of the exercise I designed completely myself and half of it is borrowed from my tutor’s idea. (F, 35*, *level 4 Pilates, exercise referral)*

While others simply retained or adapted the information gained during their past qualifications or experiences.

*Once you’ve learned it, you don’t unlearn it, you bring it into, even if say the aerobic session wouldn’t necessarily have an evidence base because I don’t know of any, anyone that’s done it. I certainly haven’t studied it on that level, apart from the fact that you’re making progress and you feel good. (F, 61, fitness instructor, exercise to music, exercise with older adults)*

#### Main sub-theme 1b – implementation of the chief medical officers’ physical activity guidelines into practice

Interviewees walked us through their typical instructor-client conversation; it quickly became apparent that discussions of the guidelines were often dominated by the aerobic (and balance) component(s).

*Maybe I could use it more than what I actually do…and what’s good on that is it gives you different day to day stuff that you can be doing like going out for a brisk walk or going for a cycle, playing bowls…So, I find that quite a useful graphic actually…(Male, aged 56; hereinafter, M, 56, chair-based exercise, exercise to music, gym instructor, personal trainer, exercise referral, postural stability instructor)*.

Data indicate that overall (n = 14), interviewees were not implementing any components of the CMO PAG in practice.

*Obviously, you mentioned the guidelines, I don’t necessarily use any kind of overall physical activity guidelines. (M, 29, master’s degree in sport and exercise science)*

*Well, this is all new, not to me, obviously, but my thoughts around it because you see lots of fitness instructors…yeah, no-one’s really, I feel…I think, is following those guidelines. And even in leisure centres and things, there’s no real thought about the prescription of exercise, I feel, anyway. (F, 39)*

As an exception to our overall findings, one interviewee (who became a personal trainer after retirement), described being very specific with her older adult followers on how to align their activities to meet each of the components within CMO PAG.

*So, I say, if you do your 6 K daily steps challenge and you do my class twice a week, or you find some other strengthening exercise, then you will be relatively okay. If you can do it more, great; but, as a minimum, that’s what you should be doing. And I do…I do…I do tell them that. (F, 71, level 3 personal trainer)*

#### Main sub-theme 1c – suggestions for improvements to the chief medical officers’ physical activity guidelines

There was palpable concern that communicating the minimum thresholds of the guidelines (for example, 150 min a week of moderate intensity aerobic activity and building strength on at least 2-days-a-week) to their less-abled members might overwhelm them, ‘*I think the guidelines, sometimes it’s just pitched a bit too high, you know?’ (F, 63, master’s degree in public health, chair-based yoga, chair-based exercise)*.

Another interviewee, while simultaneously shaking her head, mentions that the infographic is not at all relatable to her older adult members, ‘*There is a guy on a cross trainer, and I just think, really??’ (F, 59, level 2 fitness qualification, qualified counsellor)*.

Thus, for many exercise instructors, the CMO PAG were seen as a barrier to inspiration and motivation of older adults in achieving the minimum recommendations,

*…for them to see that as the what the current guidelines is can be quite daunting, and demotivated to think, well, that’s way out of my league initially. (M, 29, master’s degree in sport and exercise science)*

Interviewees indicated that the single category of ‘adults and older adults’ in the CMO PAG needed to be broken down further into a more nuanced set of categories,

*I would like to see categories. I would like them to breakdown specific guidelines for specific populations. One size fits all isn’t necessarily beneficial from an individual’s perspective, but also from a practitioner’s perspective (M, 29, master’s degree in sport and exercise science)*.

Separating the guidelines by ability-level was suggested as a more effective strategy than the current chronological-age category (19 years of age and older).

*I think age should be taken out and maybe, I don’t know, I’m trying to think mobility, but I don’t know, it’s a hard one to do, but I think age should be taken out, I don’t know what the answer is but it’s not age. (F, 53, level 3 older adults, exercise referral, trained as physiotherapist)*

A call for increased detail on the infographic was also discussed,

*There could be more prescription in there of that, because if a member of the general public saw that infographic, they wouldn’t really know sort of what that meant. (M, 56, chair-based exercise, exercise to music, gym instructor, personal trainer, exercise referral, postural stability instructor)*

From interviewees’ perspective, a major area for improvement was the need to educate older adults on the *activities* that build strength. Instructors reported that when older adults describe their current activities, their members often describe meeting the strength recommendations with aerobically-dominated activities.

*There’s a lack of knowledge by people that as they get older, they need to do weight bearing exercise, I mean, that is a big lack of knowledge and well people think they can just get away with a bit of running and a bit of cycling there and they’ll be fine. (F, 58, running coach, personal trainer, cycling coach, nutrition coach)*

There was also discussion around the need to educate older adults on the *benefits* of building strength as one ages.

*And I don’t think that the dots are very well joined up, therefore, in terms of people understanding the impact of the ageing process on them and how gym activity generally but specifically resistance training as well as balance and flexibility work really can benefit them. (M, 62, personal trainer, exercise referral, cancer rehabilitation)*

Interviewees also expressed some concern that there was not enough education or signposting to ensure older adults stay safe while strength training on their own.

*Well, you don’t want to go to a class, but you could do your walk and then do a bit of strength training, but if you’re not doing it right, you’re doing more harm than good. (F, 53, level 3 older adults, exercise referral, trained as physiotherapist)*

Interviewees went on to discuss educating our health care practitioners on how important it was to discuss and prescribe strength training to their patients. Particularly topical, interviewees said that health care practitioners did not fully appreciate the benefit a strength training conversation and/or prescription had, not only to their older adult patient but also the potential to reduce to their own workload.

*There’s a lot of focus on GPs and their workload I actually, we, the physical activity practitioners, have the tool for them to decrease their workload. So, they should be using it. At the end the patient has the benefit, it’s all about patient benefits and if those patients are benefiting and, therefore, providing less burden on the health service and on primary care in particular then everybody’s winning. (M, 62, personal trainer, exercise referral, cancer rehabilitation)*

Other interviewees working in the referral pathway agreed that health care practitioners were not discussing, referring, or prescribing strength training at meaningful rates.

It was suggested that ideally, the CMO PAG would be updated to provide more detail for the exercise professional. That way, all older adults would receive consistent evidence-based strength training prescription. Otherwise, interviewees indicated a more deeply rooted problem in that instructors were not adequately prepared following their qualification to prescribe strength training to older adults.

*But does it say…does it say how to [prescribe strength training]? Sorry, does it say how to do that? (F, 71, level 3 personal trainer)*

*Yeah, that could be strength to do very little or strength to do too much, it’s not specific enough to say, this is what it is, yeah. Especially for someone who’s just newly qualified. (F, 39, bachelor’s degree in sport, level 3 exercise referral)*

However, interviewees did acknowledge that the details they need to prescribe evidence-based strength training, whilst not readily available in the CMO PAG, could be sourced elsewhere. They also admitted that omitting these details may cause exercise instructors to source their strength training information from another (potentially unreliable) source, increasing the risk of providing a programme that at best, lacks consistency across instructors and at worse, lacks efficacy and contributes to a loss of strength training adherence.

*Things like the guidelines, you know, it’s all written down somewhere, they can all find it, we can all find it, but they’re not going to. (F, 56, Zumba, chair-based exercise)*

The importance was not just on improving the CMO PAG, but in improving its awareness and usage. A number of interviewees spoke passionately about the topic and expressed a direct call to action to the interviewer. Exercise instructors wanted researchers to bring about change with their research, especially research that would help to remove some of the barriers they face in practice. The barriers presented included educating the general practitioner of the benefits of exercise referrals,

…*so realistically, what the research physiologist needs to be doing is highlighting this to the primary care practice because there is a huge amount that exercise can do for these patients if they are given the opportunity to do it and they’re only given the opportunity to do it if the primary carer decides to do it based on high quality evidence that they’ve been presented with. (M, 62, personal trainer, exercise referral, cancer rehabilitation)*

As well as barriers in the public messaging of the strength guidelines to older adults,

*We have got a lot of work to do, and it is work to be done. I am so delighted you are doing what you are doing. Because we absolutely need to change the message. (F, 59, level 2 fitness qualification, qualified counsellor)*

### Major theme 2 – building strength

#### Main sub-theme 2a – benefits of building strength

Interviewees noted that during their typical encounters, older adults were largely unaware of the *benefits* of building strength.

*No, they don’t, I think they know they need to be here but they’re not quite sure, but that’s about as far as it goes. (M, 62, personal trainer, exercise referral, cancer rehabilitation)*

Interviewees went on to describe *how* they attempt to educate their older adult members on the benefits of building strength. Despite the myriad of possible benefits of building strength, the discussions were quite the opposite. Conversations between instructor and older adult seemed to focus on having (or building) just enough strength to carry out basic activities of daily living,

*I feel we need to be strong for daily tasks for daily life and that’s the strength that you need. Well, for me, personally, I don’t feel you need masses of strength ’cause why do we need that, what’s the purpose? (F, 39, bachelor’s degree in sport, level 3 exercise referral)*

#### Main sub-theme 2b – reactions to building strength

Despite the best intentions by interviewees, discussions with older adults about becoming more physically active were regularly met with negative reactions.

*I always start with them I say, right we are going to do some exercise now and the look of horror. The hardest thing in my job is getting that initial conversation right. That is the biggest, of everything I do, that is the hardest thing. (F, 59, level 2 fitness qualification, qualified counsellor)*

Misconceptions and misperceptions about the *ability* to build strength in later life were common,

*[Older adults] don’t think they have got the potential to build strength and they are always surprised when I talk to them about it. They really don’t think that they have got the potential to do. They just think I am old, and this is how it is. (F, 59, level 2 fitness qualification, qualified counsellor)*

The misperceptions about *how* to build strength were met with even more skepticism, bordering on fear. There was fear of using a lot of weight, *‘when I first talk about strengthening exercises, they say ‘oh so we’re going to use lots of weights?’ (F, 35, level 4 Pilates, exercise referral)*.

As well as fear around using heavy weight, *‘I don’t mind doing some weights, but I don’t want to do anything really heavy.’ (F, 56, Zumba, chair-based exercise)*.

#### Main sub-theme 2c – reflections to the reactions of building strength

It was clear that quite a lot of reflection took place around what an exercise instructor should or should not be saying in an attempt to better engage older adults with strength training. The general conclusion was to avoid certain words or phrases that might intimidate their members. As such, marketing to the older adult was mentioned as a challenge, as older adults seemed to be intimidated by a number of words that may help to describe their services,

*Yes, it’s not ‘lifting weights’, it’s not ‘building’…’bulking up’ or anything like that… (F, 61, fitness instructor, exercise to music, exercise with older adults)*.

Even the mention of ‘exercise’ was discussed as having a negative connotation in the older adult cohort. Therefore, other words were often experimented with,

*I mean, I think there are quite a lot of barrier terms that create barriers. Like, I don’t think ‘exercise’ is a very good one. But it’s difficult because it is exercise. I try and use ‘physical activity’ when I can. (F, 71, level 3 personal trainer)*

Imagery through word associations posed another challenge for instructors. The perceived physical outcomes as a result of strength training were often mentioned as a deterrent to participation by their older adult members,

*[‘Strength’] is just an immediate association with someone with rippling muscles. (F, 59, level 2 fitness qualification, qualified counsellor)*

### Major theme 3 – the interpretation of evidence-based strength training

#### Main sub-theme 3a – a more engaging model than evidence-based strength training?

Interviewees believed that the prescriptive model of ‘build strength on at least 2-days-a-week’ was the reason older adults do not or cannot seem to adhere to strength training recommendations. Thus, interviewees had begun incorporating more social and engaging models of exercise – at the expense of programming with an effective dose.

*I think probably, and I’m not saying it’s the right way to do, but I’ve probably gone more down the social sort of side of things rather than it being purely just about the physical side, so try and engage with people, it’s more than just a physical activity session for older people, because that could be a turn-off. (M, 56, chair-based exercise, exercise to music, gym instructor, personal trainer, exercise referral, postural stability instructor)*

A couple other interviewees believed that their model of social/community-based prescription was a more engaging model than the evidence-based strength training model and have completely swapped ‘build strength’ for activities such as gardening or ‘*walk and talk programmes*.’

*I used to use a gym-centric model. My model now is community based. I have really just stepped away from gyms. I still use them, they are a tool. But I go out now into extra care schemes which is where I am mostly working with older adults. I am running some outside community projects, walk and talk programmes. (F, 59, level 2 fitness qualification, qualified counsellor)*

#### Main sub-theme 3b – misconceptions of evidence-based strength training

Although we cannot be certain that the lack of detail on the strength component embedded within the CMO PAG is directly contributing to misconceptions of what evidence-base strength training looks like in practice, the lack of detail is not *helping* exercise instructors translate evidence-based guidelines into practice.

*Yeah, um, they don’t kind of say you’ve got to do 30 min every day or 150 a week or anything [like the aerobic guidelines do]. (F, 63, master’s degree in public health, chair-based yoga, chair-based exercise)*

In several instances, leisure-time physical activity or other modalities of exercise seemed to be mistaken for evidence-based strength training. Here, the interviewee talks of low-effort muscular endurance activity as her strength training prescription,

*So, you know, like when we do our sitting down marching, we used to count to 20 and now we’re up to 40. So, we’re just kind of increasing their strength and in that sort of way really. (F, 63, master’s degree in public health, chair-based yoga, chair-based exercise)*

Here, the interviewee discusses getting outdoors and socialising when asked about their strength prescription,

*They [older adults] just get out the house and come and sit in a chair and watch us, get some vitamin D, social and that kind of thing. Yes, yeah. And I’d never tried it before (F, 39, bachelor’s degree in sport, level 3 exercise referral)*.

Finally, misconceptions seemed to be perpetuated by a lack of evidence-based discussions even in qualification training, professional development seminars, and during conferences.

*I sat in on a webinar that was held by a national body of instructors, but the guest speaker put herself across as an expert. So, I was pretty horrified to hear that her experience of it was caring for her father who’d had a stroke. She said, we’re ALL instructors on this webinar, she just put it out there that you didn’t really need to be qualified, you just need a lot of common sense to work with older people. (F, 61, fitness instructor, exercise to music, exercise with older adults)*

#### Main sub-theme 3c – ageism in strength and strength training

For older adults to ‘buy into’ the need for and benefits of strength and strength training, it was recognised that as a society, we need to combat (self-) ageism, and promote self-efficacy.

*There’s still kind of a negative impression to the ageing process in this country, I think probably globally. The idea that once you pass 60, you’re past [the ability to build strength]. (M, 62, personal trainer, exercise referral, cancer rehabilitation)*

Paradoxically, there was also indication of misplaced concern or ‘compassionate ageism’ throughout the interviews. There were expectations of what older adults could, should or should not do based on their assumed vulnerability that categorically occurs with increasing chronological age. For instance, strength exercises were perceived by some as too challenging for older adults,

*Well, squats is maybe not a very good example because they’re quite hard. I mean, for example, I do one exercise where I just life one arm up and the other arm comes behind and then I alternate with doing that, with straight arms. (F, 71, level 3 personal trainer)*

There was also a perceived negative effect of strength training on ageing joints,

*I’m not necessarily going to prescribe them to come in and do one specific resistance training session. It’s quite hard on the joints. The specific activities that we prescribe, for example, going up and down maybe three or four steps, they’ve got the resistance activities in there rather than prescribing specific resistance programmes for individuals that are over the age of 65. (M, 29, master’s degree in sport and exercise science)*

And a perceived increased chance of injury for older adults performing strength training,

*I don’t want to be known for ‘extreme fitness’, I want to be known for the good things that we do, not cause an injury. (F, 39, bachelor’s degree in sport, level 3 exercise referral)*

## Discussion

The aim of the study was to explore whether exercise prescription for older adults is aligned with the recommendations within the strength component of the Chief Medical Officers’ physical activity guidelines.

First, most (n = 9), not all (n = 6) exercise instructors are aware of the UK’s Chief Medical Officers’ physical activity guidelines (CMO PAG). Only one participant mentioned the strength component, indicating that the strength guidelines are still the ‘forgotten guidelines’ [27]. Previous research shows that certified exercise professionals were unable to recall the general guidance for prescribing exercise from their professional body 58% of the time, irrespective of gender, age, education, experience, or certification level [28].

In addition, nearly all instructors (n = 14) are not aligning their strength prescription with the frequency recommendation of ‘*at least two days-a-week’* for their older adult clients. These findings corroborate a survey of UK-based practitioners delivering exercise to older adults with sarcopenia [24]. Despite having international consensus from their governing body that practitioners should be offering a programme with a prominent resistance component, resistance training was the main focus of the programmes only 9% of the time and was missing from their prescriptions *entirely*, 35% of the time [24, 29]. The same survey also indicated that 65% of the time, supervised exercise was offered once-a-week or less [24]. In an audit of NHS instructors, this once-a-week offering was found to be even more common − 86% of the time [30]. While once-a-week strength training to failure has been shown to increase strength [31], failure to follow the ‘at least two days-a-week’ recommendation within the guidelines is problematic as an insufficient exercise dose for those older adults not training to failure will likely not be able to achieve the goal of building muscular strength [32]. An ageing population lacking sufficient or optimal levels of muscular strength may be increasingly susceptible to early-onset (multi-)morbidity, resulting in unsustainable economic and healthcare stress.

Our research also shows that instructors are looking to a variety of other information sources to create their strength prescription. While some of these sources may draw on the CMO PAG, not all sources mentioned would necessarily be classified as high-quality evidence [33]. Although this finding may be due to the heterogenous nature of our sample, our sample seems to match the inconsistency in job titles, roles, and qualifications found within exercise provision in the UK [34]. We build upon the concern raised by Crozier et al. (2022) by showing that not only do inconsistency in qualifications (and training) exist (as shown in our participant demographics, and particularly evidenced within our ‘misconceptions of strength training’ theme), but that this inconsistency manifests as noteworthy deficits in awareness, knowledge, and implementation of evidence-based strength prescription in routine practice.

The CMO PAG are guidelines and not full exercise prescriptions. However, the lack of detail (that is, providing only frequency and type) and tailored guidance within the strength component of the CMO PAG may partially explain why strength uptake and adherence is far less likely than its aerobic counterpart. In addition, the lack of intensity of effort and session time guidance within the strength component may oblige our instructors to look elsewhere for this information. In the best case scenario, this may lead to an individual interpretation of what evidence-based strength prescription looks like in practice, and in the worst case, this leads to a low and ineffective exercise dose. For instance, we have seen interviewees leave the load and/or intensity of effort up to their older adults, or intentionally leave it out of their prescription altogether. In some cases, exercise instructors believe that load and/or intensity of effort are not fundamental aspects of an exercise programme or believe that their members will not be able to achieve progression [22, 24, 35]. Leaving key exercise variables up to older adults is not an optimal strategy in building muscular strength, as we know that older adults will often choose the lowest load or intensity of effort available and rarely challenge themselves or their capacity without expert supervision and motivation to do so [17, 36]. Furthermore, leaving load or intensity of effort up to older adults to self-monitor and modify appropriately implies that they understand what this entails, which is likely not the case [17]. Even in a supervised programme, where the steps of resistance band progression are explicitly embedded within the evidence-based exercise protocol, less than one-quarter of older adults were found to have achieved the expected strength progression [22]. Not only do older adults report that the motivation to push themselves in a class is why they travel to see an expert instructor in the first instance, but that this personalised attention from an exercise instructor has been shown to promote continued exercise adherence [17, 37].

The suggestions to improve the CMO PAG include a clear need for definitions and quantification of important strength training variables for the exercise instructor as well as discussions surrounding language, messaging, and behaviour change techniques shown to be effective with older adults [38–40]. In addition, both exercise instructors and older adults agree that there is need to diversify the guidelines to motivate and optimise the strength of more active older adults while not demotivating those in-transition or clinically frail older adults, who will need to work their way up to the minimum recommended thresholds [17, 38, 41]. On the other hand, since load and intensity of effort are entirely scalable to individual ability in the prescription of strength training, this may act as a perceived barrier rather than actual barrier to evidence-based strength training participation.

This study adds to the growing body of evidence in the UK demonstrating poor fidelity in practice to evidence-based strength programmes, failing to optimise the benefits that strength training has for the older adult population [22, 24, 30]. However, because exercise instructors are in an important position in older adults’ lives (meeting older adults at least once per week, every week, for at least eight to 12 weeks), it is crucial that exercise instructors implement evidence-based guidelines in practice, to help address the strength training participation gap in the older adult population. The number of encounters that instructors have with older adults are greater than the number of encounters older adults have with their healthcare practitioner, and certainly more than with research teams or policy makers.

Our study has limitations. We recruited a small, heterogenous (in age, education, and experience), convenience sample (n = 15) of self-identified exercise instructors in the UK. Respondents to the study advertisement may have a particular interest in sharing their experiences with prescribing exercise. This research was carried out with instructors with varying ages, career paths, education, qualifications, and experiences, so our results may not be specific to all exercise instructors who work with the older adult population but our sample is likely generalisable given the current landscape of instructors and exercise provision in the UK [34, 42]. We noticed that many instructors classified themselves as ‘older adults,’ which is not uncommon for those that choose to work with this population [42]. We also noticed that most instructors worked with older adults who may be classified as ‘in-transition’ or clinically frail, rather than robust older adults. This may indicate that there is a lack of instructors who are interested in, or comfortable working with the younger, more active older adult population. On the other hand, the strength of this study lies in the depth of conversations had with 15 exercise instructors with diverse educational backgrounds and experiences with older adult clientele. The rich data we have collected in the current study has been immediately verified by using individual, reflexive follow-up questions with each participant, as opposed to potentially misinterpreting conclusions that could result from collecting this data using other research methods (for instance, online surveys with dichotomous answer suggestions).

## Conclusion

Despite the strength training component being embedded within the UK’s CMO PAG for over a decade now, there is minimal evidence that strength training programmes are underpinned by their recommendations. From these interviews, the strength training programmes described are likely not optimised for strength gain, and other important benefits such as the prevention of age-associated disability and disease. Future research should look to quantify this statement by objectively measuring muscular strength in older adults throughout their participation in community classes. Despite ample academic evidence in this area, there is a severe lack of emphasis on load, intensity of effort, and progression in practice [43]. We suggest (i) further detail on *how* to build strength within the guidelines (ii) a handbook to detail the intended implementation of the CMO PAG in practice, (iii) an update to theoretical and practical teaching materials and courses, and/or (iv) a re-(education) of exercise instructors (perhaps through commitment to ongoing CPD) already in the field. These suggestions may be necessary to bring about strength training implementation that more closely aligns with the academic evidence and ensures the best possible health and longevity outcomes for an ageing population.

An iteration of the UK’s CMO PAG, could have the potential to prove useful for exercise instructors prescribing evidence-based strength training to older adults and improve the uptake and adherence of the population and contribute to national physical activity goals. To make the strength guidelines clear, evidence-based, and more inclusive, we suggest the following.

(i) a greater emphasis on intensity of effort (that is, moderate to vigorous intensity, measured using a rating of perceived exertion (RPE) scale), keeping in mind there are a number of ways to increase intensity of effort (see Fig. 1 in [44]). Inclusion of an intensity of effort scale would allow the differential programming for active older adults, older adults in transition, and clinically frail older adults,
(ii) a greater selection of exercises and the removal of low intensity of effort activities/modalities, such as carrying bags and yoga, at least for ‘active’ older adults.
(iii) a mention of the progression principle, for example, ‘every 4–6 weeks include a gradual increase in training stress (through load, volume, duration, frequency, etc.) for continued muscular strength gain’ [43, 45].

Since higher levels of muscular strength appear to reduce the risk of age-associated disabilities, diseases, and all-cause mortality, we must continue to promote effective exercise stimuli, mitigating the accelerated loss of muscular strength in ageing populations [43]. It is our hope that this paper will continue to draw attention to, and drive practical change in the way researchers, policy makers, and governments support the creation, dissemination, and implementation of the physical activity guidelines to the people who are responsible for prescribing strength training to the older adult population [46].

## Data Availability

The anonymised datasets used and/or analysed during the current study available from the corresponding author on reasonable request.
